# Supplementary figures and images for: DNA methylation and gene expression profiling reveal potential association of retinol metabolism related genes with hepatocellular carcinoma development
Source: PeerJ. 2024 Aug 23;12:e17916. doi: 10.7717/peerj.17916 (PMC11348899; doi:10.7717/peerj.17916)

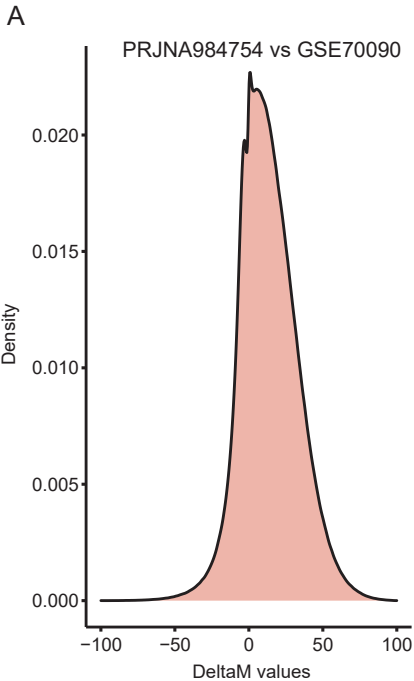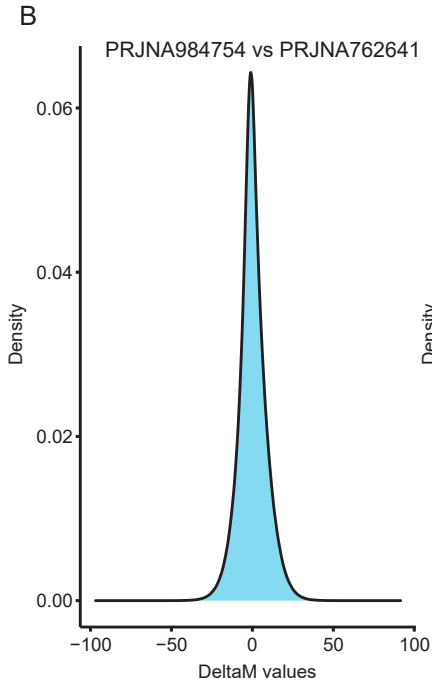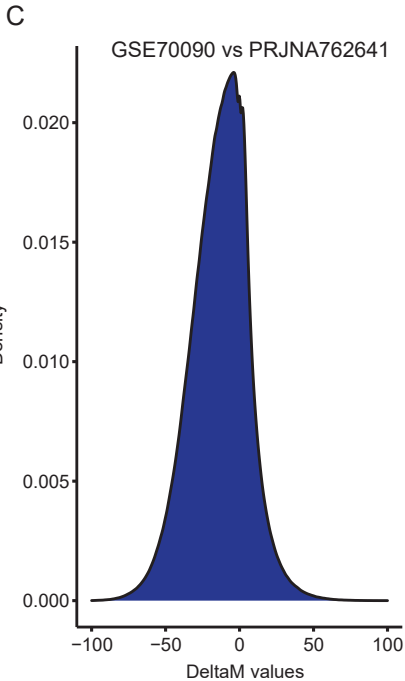

Supplement: Figure S1 — (A) The comparison of PRJNA984754 vs. GSE70090. (B) The comparison of PRJNA984754 vs. PRJNA762641. (C) The comparison of GSE70090 vs. PRJNA762641. Y-axis is the density, and x-axis is the difference in methylation values (deltaM) of CpGs between two datasets, ranging from −100 to 100 (percentile). When two datasets are identical, deltaM should be 0 (corresponding to one peak at position 0). [file peerj-12-17916-s001.pdf]

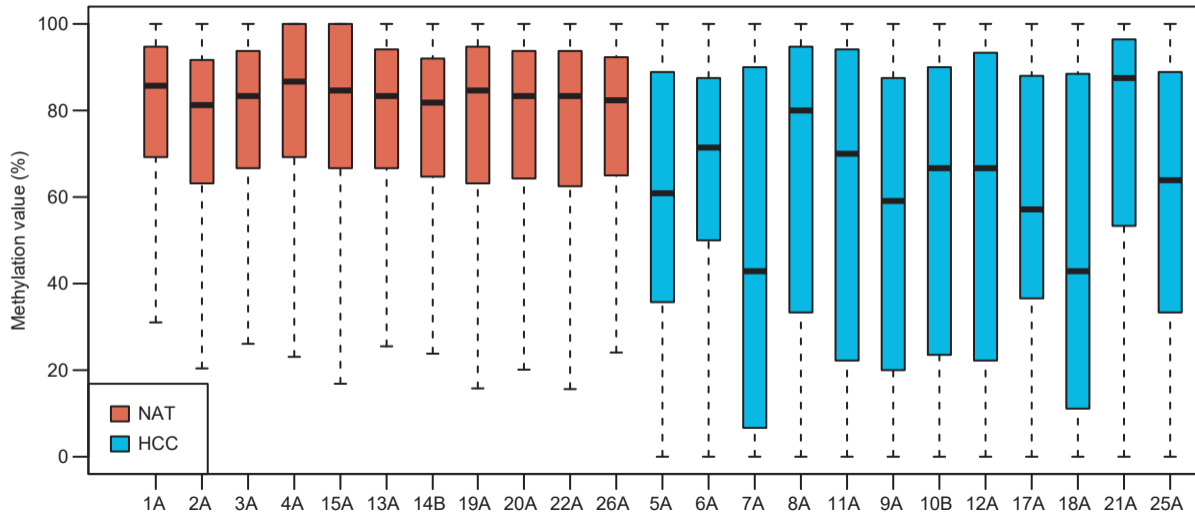

Supplement: Figure S2 — One sample (16A) was excluded because of low sequencing depth. [file peerj-12-17916-s002.pdf]

A

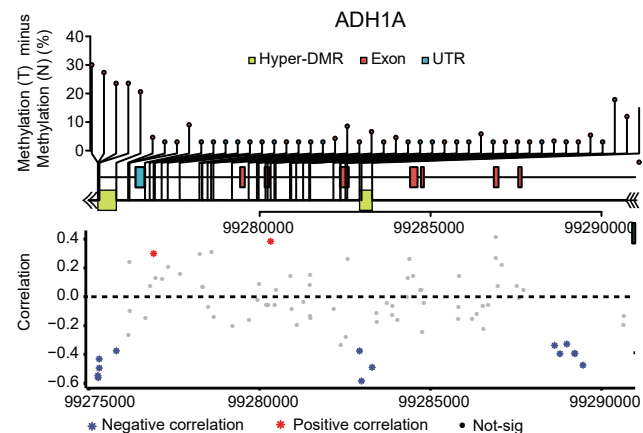

B

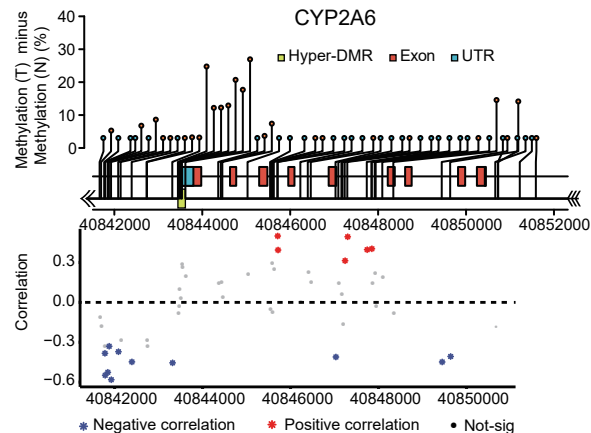

C

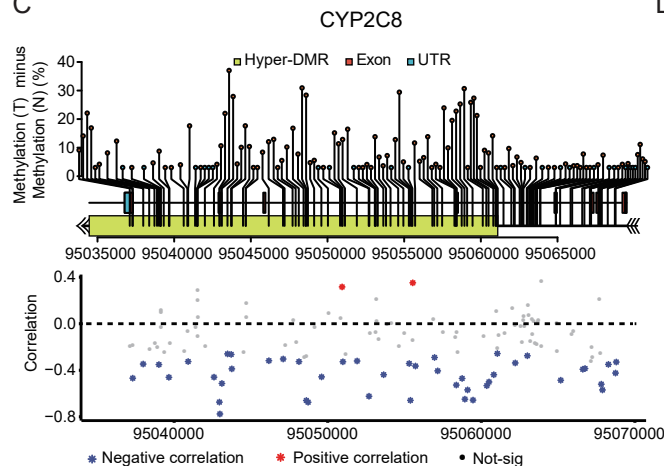

D

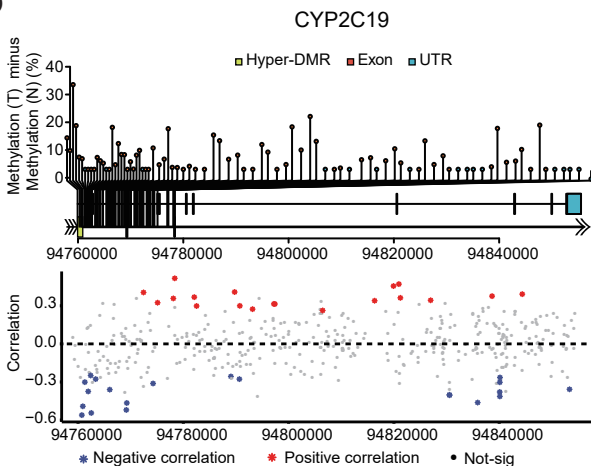

Supplement: Figure S3 — The upper part of each panel shows the DMRs, exons/UTRs, and CpGs within the gene. Y-axis indicates the methylation values of CpGs between HCCs and NATs. The lower part of each panel shows the correlation of CpG methylation with gene expression. Red stars represent significantly positive correlations and blue stars represent significantly negative correlations. [file peerj-12-17916-s003.pdf]

**ADH1A (chr4\_99275265\_99275805)**

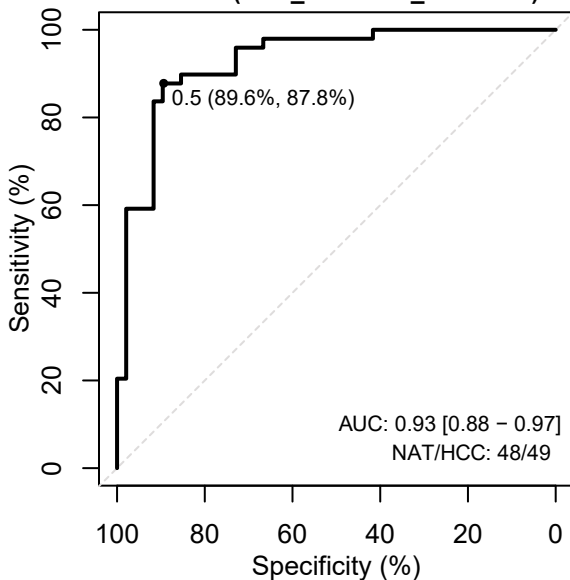

**CYP2A6 (chr19\_40843446\_40843616)**

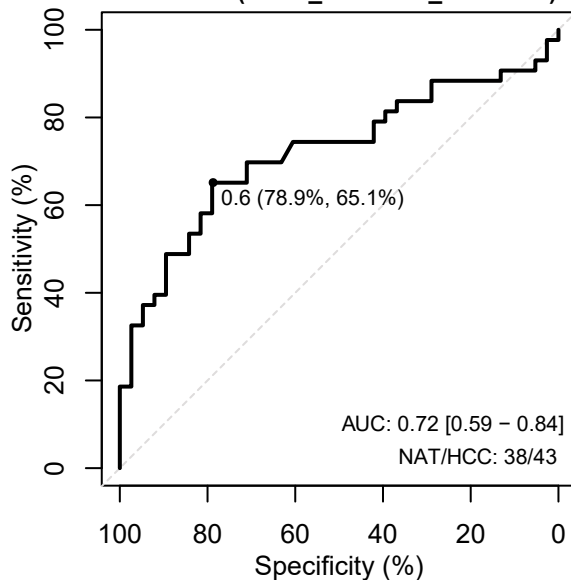

**CYP2C8 (chr10\_95034460\_95061102)**

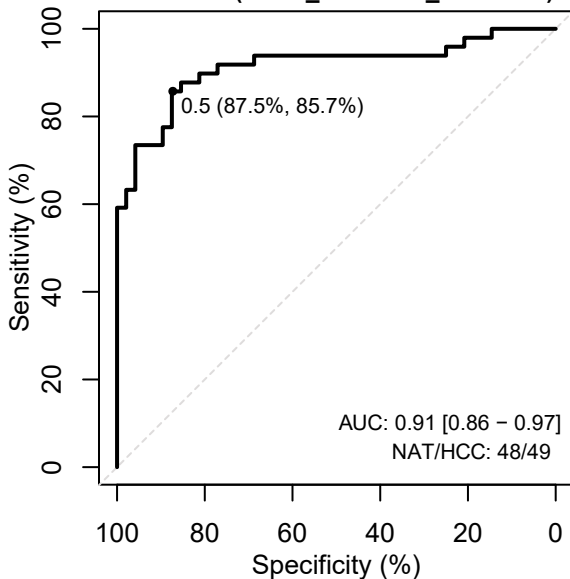

**CYP2C19 (chr10\_94759925\_94760906)**

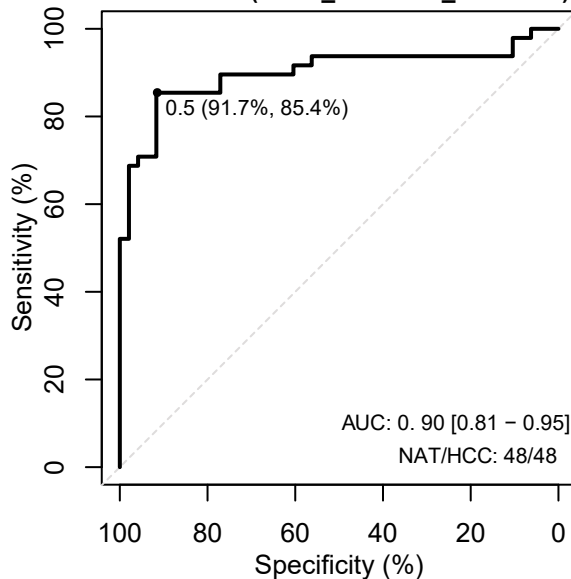

Supplement: Figure S4 — The point in each curve indicates the optimal cut-off value, and the optimal specificity and sensitivity. [file peerj-12-17916-s004.pdf]

A

## ADH1A

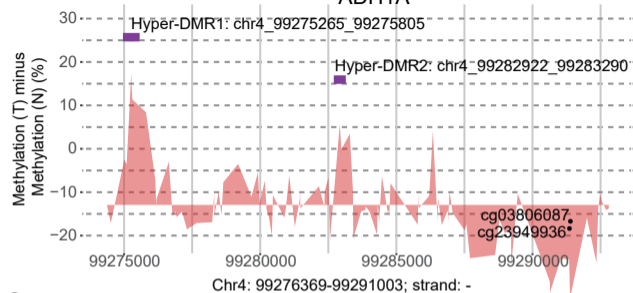

B

## CYP2A6

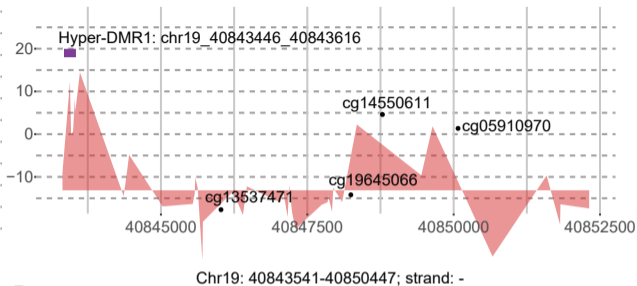

C

## CYP2C8

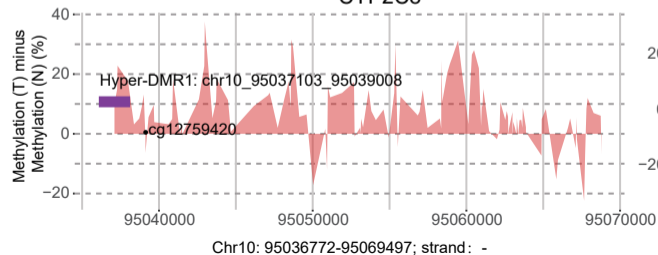

D

## CYP2C19

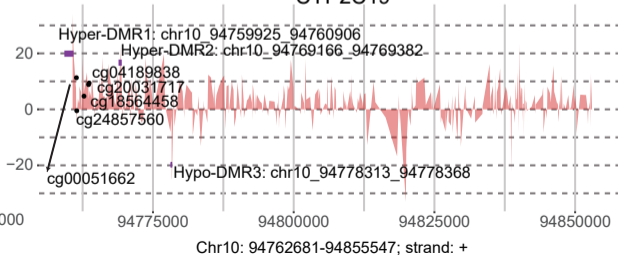

Supplement: Figure S5 — The orange areas indicate the CpGs’ methylation values determined by WGBS data and the purple rectangles indicate the location of DMRs. Each point represents a probe of 450k array. The Y-axis indicates the methylation difference between HCC and NATs. The x-axis indicates the genomic coordinates of each gene. [file peerj-12-17916-s005.pdf]

A

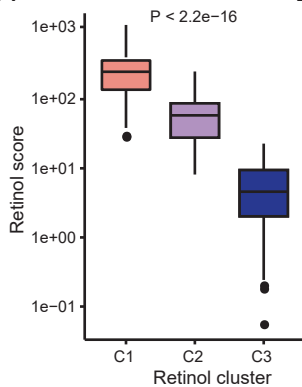

B

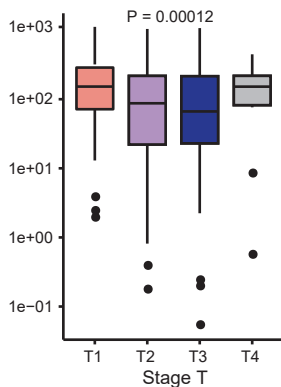

C

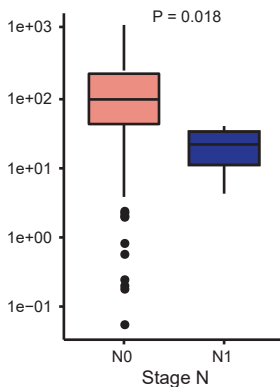

D

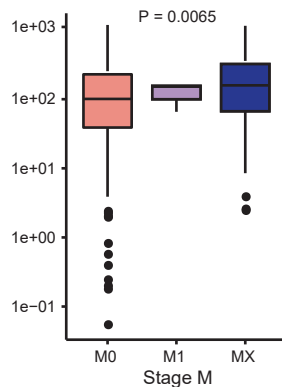

E

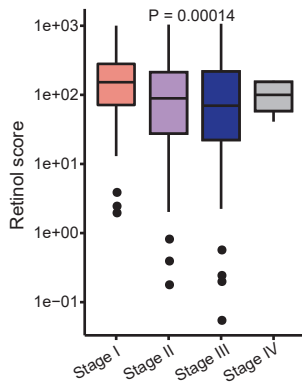

F

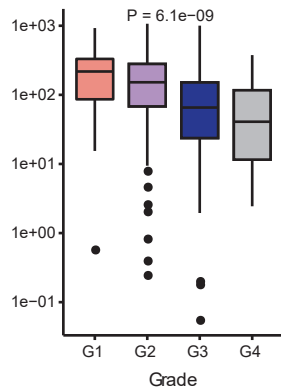

G

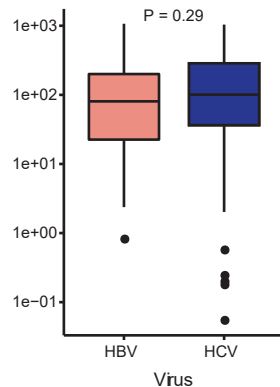

H

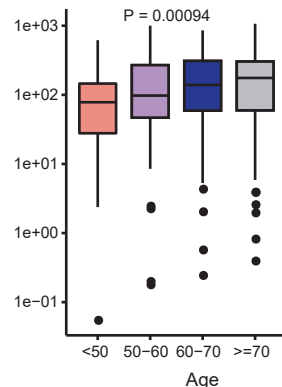

Supplement: Figure S6 — The retinol scores were calculated using the average expression values of the four genes. [file peerj-12-17916-s006.pdf]

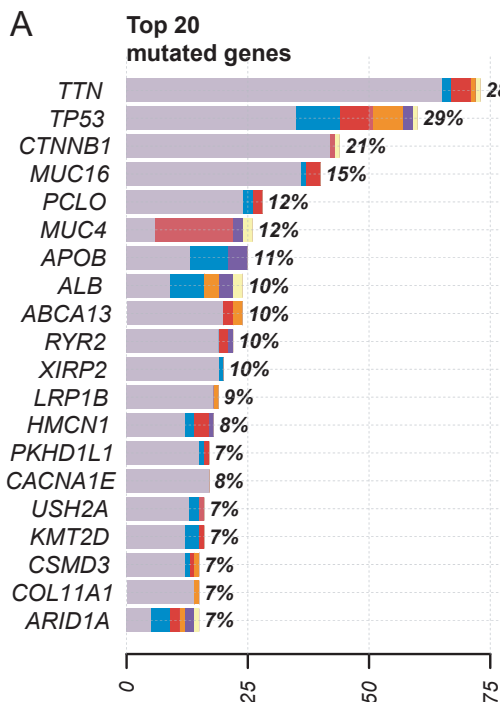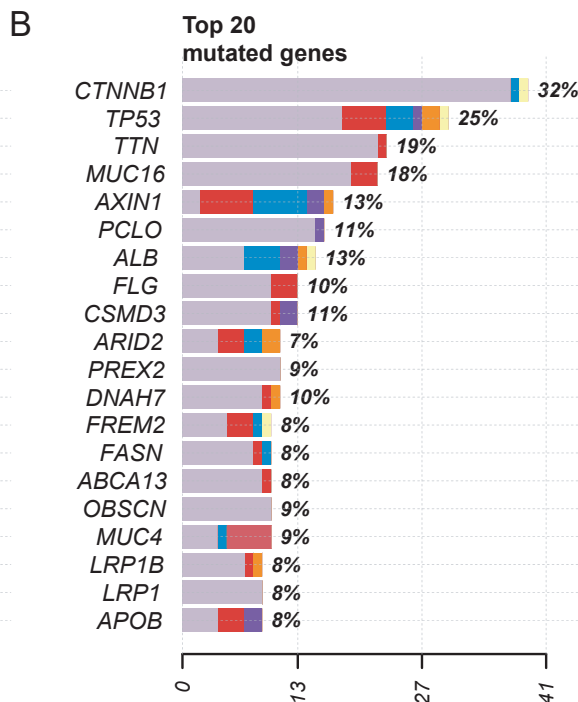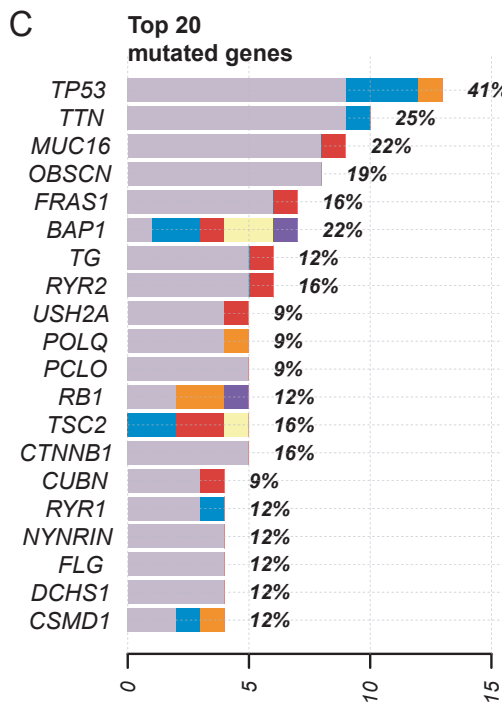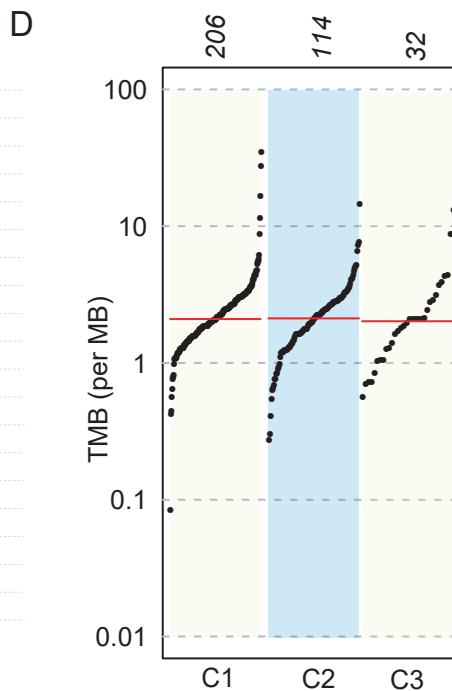

Supplement: Figure S7 [file peerj-12-17916-s007.pdf]

A

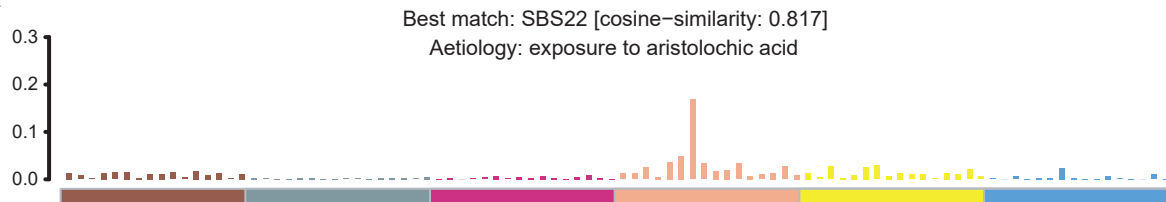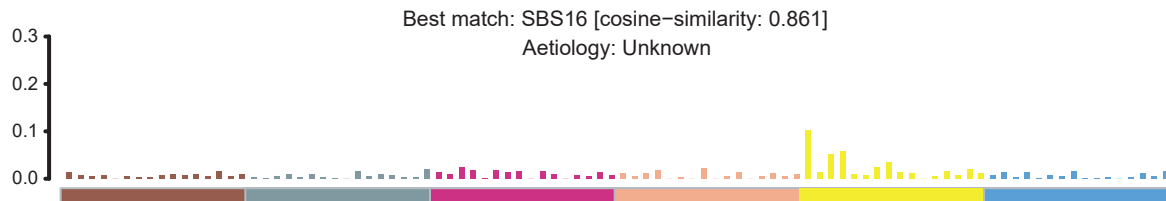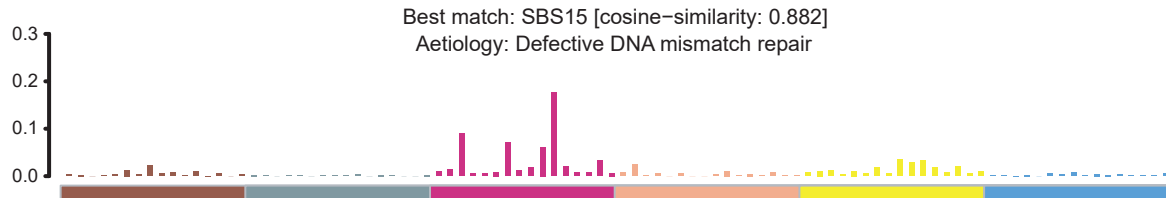

B

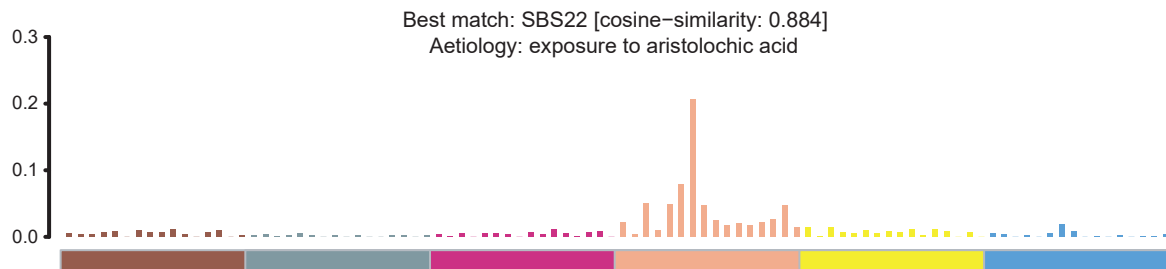

C

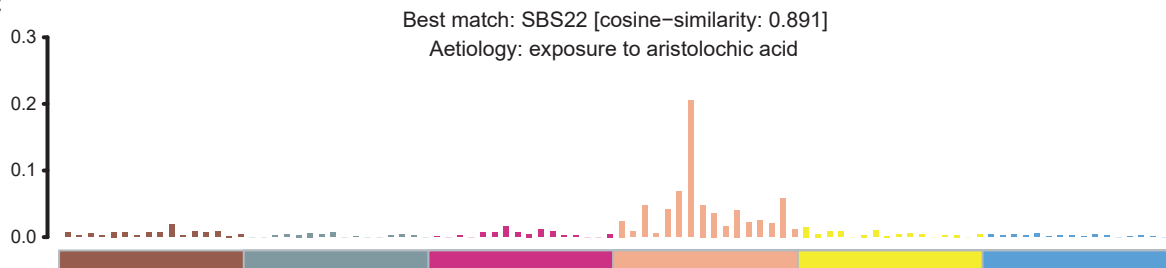

Supplement: Figure S8 [file peerj-12-17916-s008.pdf]

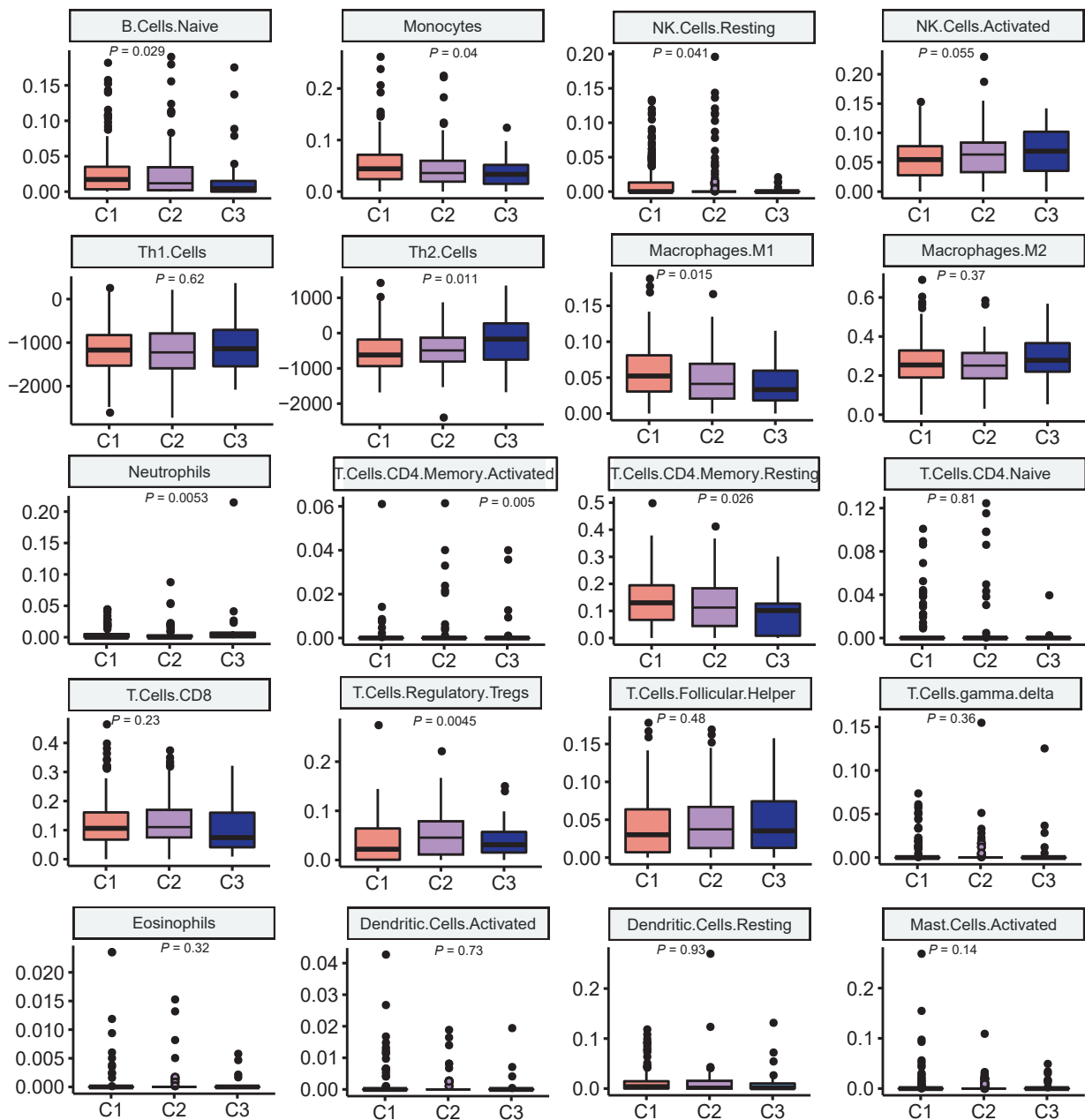

Supplement: Figure S9 [file peerj-12-17916-s009.pdf]

A

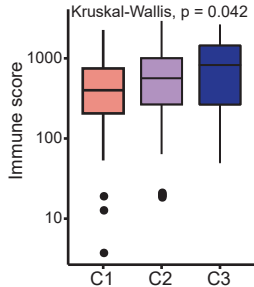

B

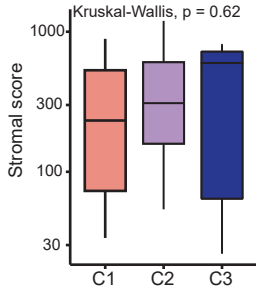

C

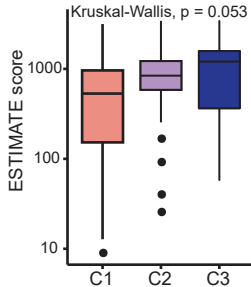

D

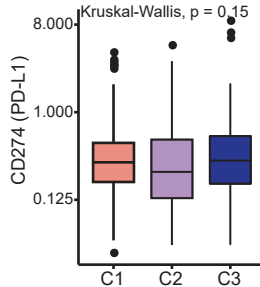

E

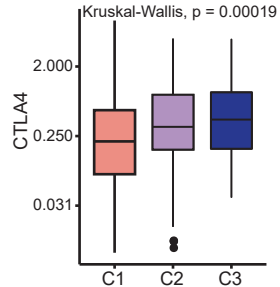

Supplement: Figure S10 [file peerj-12-17916-s010.pdf]

A

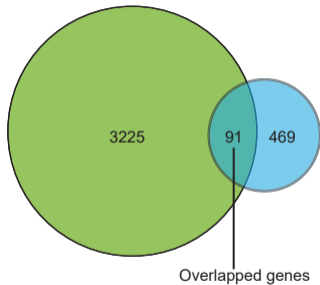

B

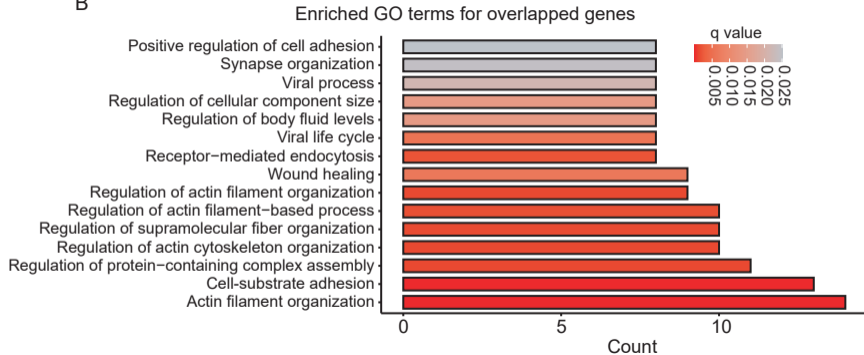

Supplement: Figure S12 [file peerj-12-17916-s012.pdf]
