# Supplementary material for: DNA methylation and gene expression profiling reveal potential association of retinol metabolism related genes with hepatocellular carcinoma development
Source: PeerJ. 2024 Aug 23;12:e17916. doi: 10.7717/peerj.17916 (PMC11348899; doi:10.7717/peerj.17916)
Supplement: Figure S11 [file peerj-12-17916-s011.pdf]

Subpopulations	B cell	BM & Prog.	Chondrocyte	DC	Embryonic stem cell	Endothelial cell	Epithelial cells	Erythroblast	Hepatocyte	Macrophage	Monocyte	Neuron	Neutrophil	NK cell	Pre-B cell CD34-	Pre-B cell CD34+	T cell
14	52	0	0	0	0	0	0	0	149	0	1	0	0	1	0	0	5
13	0	0	0	0	0	0	0	0	233	0	0	0	0	0	0	0	0
12	0	0	0	3	0	0	1	0	242	27	7	0	1	11	1	0	56
11	0	0	0	0	0	0	0	0	583	0	0	0	0	0	0	0	0
10	1	6	0	0	1	0	2	0	687	0	0	1	0	0	0	0	12
9	0	0	0	0	0	0	1	0	797	0	0	0	0	0	0	0	0
8	0	0	1	0	0	1	0	0	949	0	0	0	0	0	0	0	0
7	0	0	1	0	0	0	1	0	1108	0	0	0	0	0	0	0	0
6	0	15	0	0	1	0	4	5	1171	0	0	3	0	0	0	1	1
5	0	0	0	0	1	0	0	0	1244	0	0	0	0	0	0	0	0
4	0	0	0	0	0	0	1	0	1385	0	0	0	0	0	0	0	1
3	0	0	0	0	0	0	0	0	1416	0	0	0	0	0	0	0	0
2	0	0	0	0	0	0	0	0	1448	0	0	0	0	0	0	0	0
1	0	0	0	0	0	0	0	0	1919	0	0	0	0	0	0	0	0
0	0	0	0	0	0	0	1	0	2005	0	0	0	0	1	0	0	1

Annotated cell types
