# Supplementary material for: DNA methylation and gene expression profiling reveal potential association of retinol metabolism related genes with hepatocellular carcinoma development
Source: PeerJ. 2024 Aug 23;12:e17916. doi: 10.7717/peerj.17916 (PMC11348899; doi:10.7717/peerj.17916)
Supplement: Table S1 [file peerj-12-17916-s013.docx]

**Supplementary Table 1. The clinical characteristics of 12 HCC patients.**

| HCC ID | NAT ID | Gender | Age | Etiology | BCLC | CNLC | Tumor size(cm) | MVI | AFP (μg/L） |
| --- | --- | --- | --- | --- | --- | --- | --- | --- | --- |
| 5A | 1A | male | 76 | HBV | A | Ia | 4.2*4.1 | M0 | 2.4 |
| 6A | 2A | male | 70 | HBV | B | Ib | 5.4*4.4 | M0 | 8.98 |
| 7A | 3A | male | 75 | HBV | A | Ia | 3.9*3.7 | M0 | 10.5 |
| 8A | 4A | male | 68 | non-HBV | B | Ib | 6.2*6.1 | M0 | 1.9 |
| 11A | 15A | male | 74 | HBV | A | Ib | 1.5*2.3 | M1 | 69.1 |
| 9A | 13A | male | 55 | HBV | B | Ib | 9.0*5.3 | M0 | 13394.7 |
| 10A | 14A | male | 68 | non-HBV | B | Ib | 5.7*5.6 | M1 | 3.42 |
| 12A | 16A | male | 66 | non-HBV | B | Ib | 6.1*6.2 | M0 | 7.17 |
| 17A | 19A | male | 68 | HBV | A | Ib | 3.6*3.2 | M0 | 2 |
| 18A | 20A | male | 58 | HBV | C | IIIb | 9.2*8.9 | M1 | 5.02 |
| 21A | 22A | male | 72 | HBV | B | Ib | 5.4*4.2 | M0 | 3.81 |
| 25A | 26A | male | 52 | HBV | B | IIa | 12.3*8.1 | M1 | 100000 |
