# Supplementary material for: DNA methylation and gene expression profiling reveal potential association of retinol metabolism related genes with hepatocellular carcinoma development
Source: PeerJ. 2024 Aug 23;12:e17916. doi: 10.7717/peerj.17916 (PMC11348899; doi:10.7717/peerj.17916)
Supplement: Table S2 [file peerj-12-17916-s014.docx]

**Supplementary Table 2. Sample information for the PRJNA762641 dataset.**

| Run | Age | Isolate | Sample type | Sex | Bases | Smoking | Alcohol | HBV infection | AFP (ng/mL) |
| --- | --- | --- | --- | --- | --- | --- | --- | --- | --- |
| SRR15860791 | 70 | Patient_1 | Tumor | female | 91198875000 | No | Yes | No | 1.3 |
| SRR15860730 | 70 | Patient_1 | Normal |  | 90224615700 |  |  |  |  |
| SRR15860670 | 58 | Patient_10 | Tumor | male | 89969430600 | Yes | No | Yes | 675.9 |
| SRR15860718 | 58 | Patient_10 | Normal |  | 91783177500 |  |  |  |  |
| SRR15860789 | 72 | Patient_11 | Tumor | male | 90725457000 | Yes | cessation | No | 4 |
| SRR15860717 | 72 | Patient_11 | Normal |  | 89703478800 |  |  |  |  |
| SRR15860778 | 72 | Patient_12 | Tumor | male | 95877669300 | Yes | Yes | No | 155.8 |
| SRR15860716 | 72 | Patient_12 | Normal |  | 91660254900 |  |  |  |  |
| SRR15860767 | 69 | Patient_14 | Tumor | male | 1.07026E+11 | No | Yes | No | 0.9 |
| SRR15860714 | 69 | Patient_14 | Normal |  | 1.10654E+11 |  |  |  |  |
| SRR15860756 | 51 | Patient_15 | Tumor | male | 1.13172E+11 | No | No | Yes | 1502.5 |
| SRR15860713 | 51 | Patient_15 | Normal |  | 1.09327E+11 |  |  |  |  |
| SRR15860753 | 62 | Patient_16 | Tumor | male | 1.10466E+11 | Yes | No | Yes | 1.7 |
| SRR15860712 | 62 | Patient_16 | Normal |  | 1.07793E+11 |  |  |  |  |
| SRR15860752 | 34 | Patient_19 | Tumor | male | 1.02657E+11 | No | No | Yes | 80000 |
| SRR15860711 | 34 | Patient_19 | Normal |  | 1.09463E+11 |  |  |  |  |
| SRR15860790 | 52 | Patient_2 | Tumor | male | 91858940100 | No | No | No | 28.5 |
| SRR15860729 | 52 | Patient_2 | Normal |  | 89682432000 |  |  |  |  |
| SRR15860750 | 59 | Patient_21 | Tumor | male | 1.03221E+11 | Yes | No | Yes | 127.3 |
| SRR15860710 | 59 | Patient_21 | Normal |  | 1.07296E+11 |  |  |  |  |
| SRR15860749 | 59 | Patient_22 | Tumor | male | 1.01797E+11 | No | No | Yes | 23 |
| SRR15860708 | 59 | Patient_22 | Normal |  | 98334673800 |  |  |  |  |
| SRR15860748 | 78 | Patient_23 | Tumor | male | 91440884100 | cessation | Yes | Yes | 2.6 |
| SRR15860707 | 78 | Patient_23 | Normal |  | 99499118100 |  |  |  |  |
| SRR15860747 | 51 | Patient_24 | Tumor | male | 92405239800 | No | No | Yes | 80000 |
| SRR15860706 | 51 | Patient_24 | Normal |  | 90491207100 |  |  |  |  |
| SRR15860744 | 60 | Patient_25 | Tumor | male | 96191880300 | Yes | Yes | No | 55.9 |
| SRR15860705 | 60 | Patient_25 | Normal |  | 92294118900 |  |  |  |  |
| SRR15860743 | 54 | Patient_26 | Tumor | male | 93163680900 | Yes | Yes | Yes | 140.8 |
| SRR15860704 | 54 | Patient_26 | Normal |  | 90413921400 |  |  |  |  |
| SRR15860742 | 61 | Patient_27 | Tumor | male | 90642042900 | Yes | Yes | Yes | 9.9 |
| SRR15860703 | 61 | Patient_27 | Normal |  | 90928514100 |  |  |  |  |
| SRR15860741 | 66 | Patient_28 | Tumor | male | 94171271100 | No | No | No | 48.1 |
| SRR15860702 | 66 | Patient_28 | Normal |  | 90568508400 |  |  |  |  |
| SRR15860745 | 45 | Patient_3 | Tumor | female | 90547868700 | No | No | No | 21.2 |
| SRR15860728 | 45 | Patient_3 | Normal |  | 90124871100 |  |  |  |  |
| SRR15860740 | 44 | Patient_30 | Tumor | male | 1.04719E+11 | cessation | No | Yes | 73 |
| SRR15860701 | 44 | Patient_30 | Normal |  | 90248819400 |  |  |  |  |
| SRR15860739 | 71 | Patient_31 | Tumor | male | 1.0002E+11 | cessation | cessation | Yes | 3.7 |
| SRR15860700 | 71 | Patient_31 | Normal |  | 1.11679E+11 |  |  |  |  |
| SRR15860738 | 42 | Patient_32 | Tumor | male | 1.04754E+11 | No | No | Yes | 3.2 |
| SRR15860699 | 42 | Patient_32 | Normal |  | 1.01749E+11 |  |  |  |  |
| SRR15860737 | 66 | Patient_33 | Tumor | male | 1.01756E+11 | No | No | Yes | 1.9 |
| SRR15860697 | 66 | Patient_33 | Normal |  | 1.06202E+11 |  |  |  |  |
| SRR15860736 | 60 | Patient_34 | Tumor | male | 1.01923E+11 | Yes | No | Yes | 6.3 |
| SRR15860695 | 60 | Patient_34 | Normal |  | 1.03478E+11 |  |  |  |  |
| SRR15860735 | 57 | Patient_35 | Tumor | male | 1.01279E+11 | No | No | Yes | 402.8 |
| SRR15860694 | 57 | Patient_35 | Normal |  | 1.03517E+11 |  |  |  |  |
| SRR15860733 | 65 | Patient_36 | Tumor | female | 1.01917E+11 | No | No | Yes | 1.2 |
| SRR15860693 | 65 | Patient_36 | Normal |  | 1.05591E+11 |  |  |  |  |
| SRR15860732 | 50 | Patient_37 | Tumor | male | 1.07509E+11 | No | No | Yes | 9623.1 |
| SRR15860692 | 50 | Patient_37 | Normal |  | 1.09904E+11 |  |  |  |  |
| SRR15860734 | 61 | Patient_4 | Tumor | male | 90782114400 | Yes | No | Yes | 3.2 |
| SRR15860727 | 61 | Patient_4 | Normal |  | 90731449800 |  |  |  |  |
| SRR15860731 | 66 | Patient_40 | Tumor | male | 1.06644E+11 | No | Yes | No | 16 |
| SRR15860691 | 66 | Patient_40 | Normal |  | 1.04618E+11 |  |  |  |  |
| SRR15860722 | 64 | Patient_5 | Tumor | male | 90527680500 | No | No | Yes | 13171.3 |
| SRR15860726 | 64 | Patient_5 | Normal |  | 91512333300 |  |  |  |  |
| SRR15860709 | 33 | Patient_6 | Tumor | female | 96155670300 | No | No | Yes | 506.8 |
| SRR15860725 | 33 | Patient_6 | Normal |  | 90912235800 |  |  |  |  |
| SRR15860698 | 57 | Patient_7 | Tumor | female | 91833628800 | No | No | Yes | 8489.1 |
| SRR15860724 | 57 | Patient_7 | Normal |  | 91725766800 |  |  |  |  |
| SRR15860690 | 64 | Patient_8 | Tumor | male | 96426382500 | No | cessation | Yes | 1516.1 |
| SRR15860721 | 64 | Patient_8 | Normal |  | 91358631000 |  |  |  |  |
| SRR15860681 | 58 | Patient_9 | Tumor | female | 90144334800 | No | No | Yes | 28.8 |
| SRR15860719 | 58 | Patient_9 | Normal |  | 98676525600 |  |  |  |  |
