# Supplementary material for: DNA methylation and gene expression profiling reveal potential association of retinol metabolism related genes with hepatocellular carcinoma development
Source: PeerJ. 2024 Aug 23;12:e17916. doi: 10.7717/peerj.17916 (PMC11348899; doi:10.7717/peerj.17916)
Supplement: Table S3 [file peerj-12-17916-s015.docx]

**Supplementary Table 3. Sample information for the GSE70090 dataset.**

| Run | tissue | Sample type | Bases |
| --- | --- | --- | --- |
| SRR2074675 | liver | normal | 86599137400 |
| SRR2074677 | liver | tumor | 85165521600 |
| SRR2074679 | liver | normal | 81525699600 |
| SRR2074681 | liver | tumor | 124021464600 |
| SRR2074683 | liver | normal | 91290912600 |
| SRR2074685 | liver | tumor | 93067977800 |
| SRR2074687 | liver | normal | 81209715000 |
| SRR2074689 | liver | tumor | 65930836000 |
