# Supplementary material for: DNA methylation and gene expression profiling reveal potential association of retinol metabolism related genes with hepatocellular carcinoma development
Source: PeerJ. 2024 Aug 23;12:e17916. doi: 10.7717/peerj.17916 (PMC11348899; doi:10.7717/peerj.17916)
Supplement: Table S4 [file peerj-12-17916-s016.docx]

**Supplementary Table 4. Details of all datasets used in the study.**

| Datasets | Sample size | Normal | Cancer | Data type | Source |
| --- | --- | --- | --- | --- | --- |
| PRJNA984754 | 24 | 12 | 12 | WGBS | Custom |
| GSE70090 | 8 | 4 | 4 | WGBS | GEO |
| PRJNA762641 | 66 | 33 | 33 | WGBS/RNA-seq | GEO |
| TCGA-HCC | 415 | 50 | 365 | WES/450k/RNA-seq | TCGA |
| ICGC-LIHC | 439 | 202 | 237 | RNA-seq | ICGC |
| GSE151530 | 25 |  | 25 | scRNA-seq | GEO |
