# Supplementary material for: DNA methylation and gene expression profiling reveal potential association of retinol metabolism related genes with hepatocellular carcinoma development
Source: PeerJ. 2024 Aug 23;12:e17916. doi: 10.7717/peerj.17916 (PMC11348899; doi:10.7717/peerj.17916)
Supplement: Table S5 [file peerj-12-17916-s017.docx]

**Supplementary Table 5. The quality of WGBS data for 24 samples.**

| Sample ID | Total reads | Total bases | Coverage | Mean depth | Mean baseQ | Mean MapQ |
| --- | --- | --- | --- | --- | --- | --- |
| 25A | 34343639.75 | 109192464.7 | 81.85 | 25.67 | 32.54 | 35.50 |
| 21A | 35191503 | 109199726.6 | 81.87 | 26.17 | 32.68 | 35.88 |
| 22A | 35060099 | 109183341.1 | 81.84 | 26.21 | 32.73 | 35.87 |
| 19A | 32888213.42 | 109202131.2 | 81.87 | 24.47 | 32.40 | 34.65 |
| 17A | 33481484.92 | 109265108 | 81.92 | 24.95 | 32.47 | 34.78 |
| 20A | 33046244.92 | 109238218.5 | 81.87 | 24.75 | 32.63 | 35.68 |
| 10B | 31038866.75 | 109069234.8 | 81.75 | 22.84 | 32.36 | 34.83 |
| 18A | 30703800.58 | 109169050.8 | 81.80 | 22.84 | 32.36 | 34.49 |
| 14B | 29184574 | 108847046.2 | 81.58 | 21.83 | 32.38 | 34.43 |
| 26A | 25132503.25 | 108536904.4 | 81.32 | 18.74 | 32.41 | 32.98 |
| 13A | 26034467 | 109011922.5 | 81.69 | 19.39 | 30.93 | 34.19 |
| 9A | 24240713.33 | 108833854.3 | 81.52 | 17.75 | 30.83 | 33.09 |
| 15A | 22240928.25 | 108816600.3 | 81.54 | 16.60 | 30.87 | 31.93 |
| 16A | 21017988.92 | 108612691.5 | 81.38 | 15.85 | 30.70 | 31.50 |
| 11A | 24250569.5 | 108862596.1 | 81.58 | 18.11 | 30.91 | 33.13 |
| 12A | 11706577.88 | 51906015.13 | 82.11 | 19.29 | 26.76 | 28.38 |
| 8A | 28636942.67 | 109219922.7 | 81.83 | 21.02 | 31.20 | 35.86 |
| 3A | 28809690.92 | 109260360.3 | 81.89 | 21.48 | 32.34 | 35.03 |
| 7A | 29089536 | 109174280.6 | 81.80 | 21.59 | 31.28 | 36.00 |
| 2A | 26790028.75 | 109075768.5 | 81.74 | 20.05 | 31.24 | 35.76 |
| 5A | 29059778.92 | 109195922 | 81.84 | 21.40 | 32.30 | 35.13 |
| 6A | 28644495.83 | 109232971.1 | 81.88 | 21.26 | 32.29 | 35.07 |
| 4A | 28663901.67 | 109044666 | 81.68 | 20.91 | 32.56 | 35.60 |
| 1A | 27984475.33 | 109133593.7 | 81.79 | 20.89 | 31.19 | 35.80 |
