# Supplementary material for: DNA methylation and gene expression profiling reveal potential association of retinol metabolism related genes with hepatocellular carcinoma development
Source: PeerJ. 2024 Aug 23;12:e17916. doi: 10.7717/peerj.17916 (PMC11348899; doi:10.7717/peerj.17916)
Supplement: Table S6 [file peerj-12-17916-s018.docx]

**Supplementary Table 6. The top 100 DMRs with the most significant differences between HCCs and NATs.**

| DMR id | CpG count | Mean DeltaB | DMR Type | Location | Gene | Gene Type |
| --- | --- | --- | --- | --- | --- | --- |
| Chr1:241135343-241135358 | 3 | -67.97 | HypoM | Innergenic | RGS7 | protein-c |
| Chr20:61523369-61523405 | 5 | -66.26 | HypoM | Innergenic | CDH4 | protein-c |
| Chr7:125282767-125282798 | 5 | -65.90 | HypoM | Innergenic | POT1-AS1 | lncRNA |
| Chr7:3848913-3848966 | 8 | -65.58 | HypoM | Innergenic | SDK1 | protein-c |
| Chr6:43577040-43577165 | 13 | -65.06 | HypoM | Innergenic | POLH | protein-c |
| Chr7:18938310-18938377 | 9 | -62.17 | HypoM | Innergenic | HDAC9 | protein-c |
| Chr3:6295388-6295476 | 8 | -61.84 | HypoM | Innergenic | AC026167.1 | lncRNA |
| Chr2:57305423-57305475 | 5 | -61.02 | HypoM | Innergenic | AC132153.1 | lncRNA |
| Chr4:186312919-186312965 | 5 | -60.85 | HypoM | Innergenic | F11-AS1 | lncRNA |
| Chr4:163986508-163986561 | 6 | -59.99 | HypoM | Innergenic | 1-Mar | protein-c |
| Chr13:99495490-99495642 | 4 | -59.85 | HypoM | Innergenic | LINC01232 | lncRNA |
| Chr13:99495490-99495642 | 4 | -59.85 | HypoM | Innergenic | TM9SF2 | protein-c |
| Chr2:195916789-195916874 | 7 | -59.77 | HypoM | Innergenic | DNAH7 | protein-c |
| Chr7:43258198-43258257 | 3 | -59.21 | HypoM | Innergenic | HECW1 | protein-c |
| Chr7:4059735-4060176 | 9 | -59.06 | HypoM | Innergenic | SDK1 | protein-c |
| Chr10:17024702-17025224 | 5 | -58.95 | HypoM | Innergenic | CUBN | protein-c |
| Chr4:125815557-125815601 | 5 | -58.78 | HypoM | Innergenic | AC104664.1 | lncRNA |
| Chr10:7254145-7254782 | 12 | -58.47 | HypoM | Innergenic | SFMBT2 | protein-c |
| Chr17:78452459-78452503 | 5 | -58.36 | HypoM | Innergenic | DNAH17 | protein-c |
| Chr15:88056892-88057325 | 8 | -58.26 | HypoM | Innergenic | NTRK3 | protein-c |
| Chr5:89902675-89902877 | 11 | -58.22 | HypoM | Innergenic | AC113167.1 | lncRNA |
| Chr1:38709192-38709237 | 7 | -58.15 | HypoM | Innergenic | AL354702.1 | pseudogene |
| Chr8:116284617-116284661 | 6 | -58.07 | HypoM | Innergenic | LINC00536 | lncRNA |
| Chr9:21971413-21971460 | 4 | 57.97 | HyperM | Innergenic | CDKN2A | protein-c |
| Chr9:21971413-21971460 | 4 | 57.97 | HyperM | Innergenic | AL359922.1 | protein-c |
| Chr15:54024640-54024863 | 9 | -57.93 | HypoM | Innergenic | UNC13C | protein-c |
| Chr5:180145279-180145422 | 7 | -57.90 | HypoM | Innergenic | RASGEF1C | protein-c |
| Chr4:90514710-90514749 | 3 | -57.80 | HypoM | Innergenic | CCSER1 | protein-c |
| Chr1:4737721-4738515 | 11 | -57.55 | HypoM | Innergenic | AJAP1 | protein-c |
| Chr7:18967921-18968734 | 22 | -57.52 | HypoM | Innergenic | HDAC9 | protein-c |
| Chr19:41982363-41982580 | 5 | -57.48 | HypoM | Innergenic | ATP1A3 | protein-c |
| Chr19:41982363-41982580 | 5 | -57.48 | HypoM | Innergenic | AC010616.1 | protein-c |
| Chr11:122865162-122865486 | 10 | -57.48 | HypoM | Innergenic | CRTAM | protein-c |
| Chr1:11497604-11497656 | 6 | -57.44 | HypoM | Innergenic | DISP3 | protein-c |
| Chr15:75120134-75120190 | 4 | -57.28 | HypoM | Downstream | Metazoa-SRP | misc-RNA |
| Chr15:75120134-75120190 | 4 | -57.28 | HypoM | Downstream | AC113208.1 | scaRNA |
| Chr13:71528621-71529005 | 7 | -57.14 | HypoM | Innergenic | DACH1 | protein-c |
| Chr2:188972704-188972719 | 4 | -57.14 | HypoM | Upstream | COL3A1 | protein-c |
| Chr1:236668227-236668916 | 15 | -57.05 | HypoM | Innergenic | ACTN2 | protein-c |
| Chr10:23746487-23746665 | 10 | -57.04 | HypoM | Innergenic | KIAA1217 | protein-c |
| Chr4:126709214-126709806 | 12 | -57.01 | HypoM | Innergenic | AC097528.1 | lncRNA |
| Chr9:21992794-21993502 | 11 | -57.00 | HypoM | Upstream | AL449423.1 | lncRNA |
| Chr9:21992794-21993502 | 11 | -57.00 | HypoM | Upstream | CDKN2B-AS1 | lncRNA |
| Chr9:21992794-21993502 | 11 | -57.00 | HypoM | Innergenic | CDKN2A | protein-c |
| Chr9:21992794-21993502 | 11 | -57.00 | HypoM | Innergenic | AL359922.1 | protein-c |
| Chr5:178952259-178952521 | 14 | -56.85 | HypoM | Innergenic | ZNF454 | protein-c |
| Chr11:120128950-120129068 | 6 | -56.78 | HypoM | Innergenic | TRIM29 | protein-c |
| Chr5:178970450-178970950 | 14 | -56.76 | HypoM | Innergenic | AC104117.3 | lncRNA |
| Chr8:126190480-126190550 | 8 | -56.74 | HypoM | Innergenic | LINC00861 | lncRNA |
| Chr5:84294102-84294179 | 8 | -56.73 | HypoM | Innergenic | EDIL3 | protein-c |
| Chr1:17773059-17773090 | 6 | -56.66 | HypoM | Innergenic | ACTL8 | protein-c |
| Chr16:149855-149861 | 4 | -56.66 | HypoM | Innergenic | HBZ | protein-c |
| Chr7:18888143-18888419 | 21 | -56.59 | HypoM | Innergenic | HDAC9 | protein-c |
| Chr19:22697450-22697528 | 8 | -56.27 | HypoM | Upstream | RPL34P33 | pseudogene |
| Chr19:22697450-22697528 | 8 | -56.27 | HypoM | Innergenic | AC024563.1 | lncRNA |
| Chr7:3012452-3015116 | 23 | -56.20 | HypoM | Innergenic | CARD11 | protein-c |
| Chr12:8680395-8680681 | 11 | -56.19 | HypoM | Upstream | RIMKLB | protein-c |
| Chr1:198796622-198798515 | 9 | -56.18 | HypoM | Innergenic | MIR181A1HG | lncRNA |
| ChrX:22307932-22308014 | 3 | -56.18 | HypoM | Innergenic | PTCHD1-AS | lncRNA |
| Chr12:128287964-128289126 | 9 | -56.16 | HypoM | Innergenic | TMEM132C | protein-c |
| Chr3:76194809-76194867 | 5 | -56.15 | HypoM | Innergenic | ROBO2 | protein-c |
| Chr7:136065758-136066301 | 9 | -56.12 | HypoM | Innergenic | AC024084.1 | lncRNA |
| Chr1:175700061-175700127 | 4 | -56.05 | HypoM | Innergenic | TNR | protein-c |
| Chr7:157547474-157549849 | 24 | -56.05 | HypoM | Innergenic | PTPRN2 | protein-c |
| Chr5:145181145-145181350 | 13 | -55.83 | HypoM | Innergenic | AC132803.1 | lncRNA |
| Chr2:1022191-1022391 | 10 | -55.78 | HypoM | Innergenic | SNTG2 | protein-c |
| Chr8:118723692-118723753 | 6 | -55.73 | HypoM | Innergenic | SAMD12-AS1 | lncRNA |
| Chr4:91274876-91274901 | 4 | -55.71 | HypoM | Innergenic | CCSER1 | protein-c |
| Chr7:158021421-158024516 | 33 | -55.67 | HypoM | Innergenic | PTPRN2 | protein-c |
| Chr2:80843468-80843708 | 15 | -55.63 | HypoM | Innergenic | AC012355.1 | lncRNA |
| Chr1:240097546-240097635 | 8 | -55.62 | HypoM | Innergenic | FMN2 | protein-c |
| Chr8:61145289-61145360 | 3 | -55.53 | HypoM | Innergenic | CLVS1 | protein-c |
| Chr7:42142602-42144518 | 17 | -55.43 | HypoM | Innergenic | GLI3 | protein-c |
| Chr8:53229652-53229881 | 10 | -55.41 | HypoM | Innergenic | OPRK1 | protein-c |
| Chr8:53229652-53229881 | 10 | -55.41 | HypoM | Innergenic | AC009646.2 | lncRNA |
| Chr19:50051161-50051253 | 8 | 55.38 | HyperM | Upstream | AC010624.3 | lncRNA |
| Chr19:50051161-50051253 | 8 | 55.38 | HyperM | Innergenic | AC010624.1 | lncRNA |
| Chr19:50051161-50051253 | 8 | 55.38 | HyperM | Innergenic | ZNF473 | protein-c |
| Chr3:558676-558883 | 9 | -55.37 | HypoM | Innergenic | LINC01266 | lncRNA |
| Chr5:112263871-112263882 | 3 | -55.33 | HypoM | Innergenic | EPB41L4A | protein-c |
| Chr4:19238791-19238811 | 4 | -55.30 | HypoM | Innergenic | LINC02438 | lncRNA |
| Chr1:177450214-177450274 | 7 | -55.28 | HypoM | Innergenic | AL136114.1 | lncRNA |
| Chr2:107747954-107748363 | 9 | -55.17 | HypoM | Upstream-Body | LINC01885 | lncRNA |
| Chr9:76849774-76850142 | 6 | -55.17 | HypoM | Innergenic | PCA3 | lncRNA |
| Chr9:76849774-76850142 | 6 | -55.17 | HypoM | Innergenic | PRUNE2 | protein-c |
| ChrX:80260675-80260707 | 3 | -55.10 | HypoM | Innergenic | CHMP1B2P | pseudogene |
| Chr19:51496195-51496427 | 11 | -55.07 | HypoM | Innergenic | SIGLEC12 | protein-c |
| Chr6:97773151-97773309 | 8 | -55.06 | HypoM | Innergenic | AL589740.1 | lncRNA |
| Chr19:38422901-38423440 | 3 | -55.06 | HypoM | Innergenic | RASGRP4 | protein-c |
| Chr18:75118614-75118874 | 7 | -54.99 | HypoM | Innergenic | AC015819.5 | lncRNA |
| Chr18:11787640-11787656 | 3 | -54.99 | HypoM | Innergenic | GNAL | protein-c |
| Chr1:186163388-186163540 | 6 | -54.98 | HypoM | Innergenic | HMCN1 | protein-c |
| Chr1:245223641-245224082 | 8 | -54.96 | HypoM | Innergenic | KIF26B-AS1 | lncRNA |
| Chr1:245223641-245224082 | 8 | -54.96 | HypoM | Innergenic | KIF26B | protein-c |
| Chr10:25466047-25466274 | 10 | -54.96 | HypoM | Innergenic | GPR158 | protein-c |
| Chr7:71821337-71823590 | 33 | -54.95 | HypoM | Innergenic | CALN1 | protein-c |
| Chr1:192207226-192207400 | 11 | -54.90 | HypoM | Innergenic | AL390957.1 | lncRNA |
| Chr17:30970923-30970967 | 4 | 54.90 | HyperM | Upstream | AC138207.4 | lncRNA |
| Chr17:30970923-30970967 | 4 | 54.90 | HyperM | Upstream | RNF135 | protein-c |
| Chr18:25224636-25225199 | 14 | -54.78 | HypoM | Innergenic | ZNF521 | protein-c |
