# Supplementary material for: DNA methylation and gene expression profiling reveal potential association of retinol metabolism related genes with hepatocellular carcinoma development
Source: PeerJ. 2024 Aug 23;12:e17916. doi: 10.7717/peerj.17916 (PMC11348899; doi:10.7717/peerj.17916)
Supplement: Table S7 [file peerj-12-17916-s019.docx]

**Supplementary Table 7. Information on the 17 hyper-downregulated genes.**

| Gene | NAT-EXP | HCC-EXP | NAT-MET | HCC-MET | Corr | CorrP |
| --- | --- | --- | --- | --- | --- | --- |
| ADH1A | 974.42 | 399.72 | 64.24 | 91.05 | -0.56 | 3.69E-06 |
| AL161668.4 | 15.96 | 2.59 | 39.23 | 65.59 | -0.75 | 1.83E-11 |
| ALB | 123431.79 | 32345.70 | 44.94 | 59.54 | -0.68 | 6.04E-10 |
| CYP2A6 | 630.55 | 139.07 | 59.11 | 76.66 | -0.31 | 1.34E-02 |
| CYP2C19 | 12.62 | 1.77 | 61.08 | 81.08 | -0.56 | 4.68E-06 |
| CYP2C8 | 718.21 | 120.59 | 69.28 | 79.98 | -0.78 | 2.16E-11 |
| ECM1 | 29.06 | 11.62 | 70.50 | 83.28 | -0.64 | 3.46E-03 |
| GADD45B | 146.66 | 55.44 | 23.71 | 35.70 | -0.27 | 1.71E-01 |
| GCDH | 72.91 | 36.12 | 57.40 | 71.00 | 0.25 | 6.47E-03 |
| GHR | 59.61 | 14.00 | 33.19 | 47.39 | -0.49 | 6.44E-03 |
| ID1 | 122.51 | 32.83 | 33.46 | 49.11 | -0.73 | 2.62E-06 |
| RNF152 | 11.17 | 3.25 | 15.45 | 26.74 | -0.53 | 5.02E-03 |
| SDS | 323.48 | 87.03 | 61.72 | 75.13 | -0.65 | 1.32E-06 |
| SPRYD4 | 25.40 | 8.93 | 61.74 | 85.58 | -0.82 | 8.13E-06 |
| SRD5A2 | 37.15 | 5.77 | 38.77 | 61.94 | -0.47 | 6.05E-05 |
| TCIM | 67.64 | 26.13 | 59.63 | 70.91 | -0.72 | 8.14E-12 |
| VMO1 | 11.62 | 5.20 | 49.88 | 60.55 | -0.48 | 2.87E-02 |
