# Supplementary material for: DNA methylation and gene expression profiling reveal potential association of retinol metabolism related genes with hepatocellular carcinoma development
Source: PeerJ. 2024 Aug 23;12:e17916. doi: 10.7717/peerj.17916 (PMC11348899; doi:10.7717/peerj.17916)
Supplement: Table S8 [file peerj-12-17916-s020.docx]

**Supplementary Table 8. Methylation values of the 12 probes associated with the four genes in TCGA HCC dataset.**

| Symbol | Probe | Tumor β | NAT β | P value | FDR | Delta β |
| --- | --- | --- | --- | --- | --- | --- |
| ADH1A | cg23949936 | 0.3384 | 0.3929 | 7.64E-04 | 1.00E+00 | -0.05451 |
| ADH1A | cg03806087 | 0.3774 | 0.4153 | 6.29E-03 | 1.00E+00 | -0.03789 |
| CYP2A6 | cg14550611 | 0.3072 | 0.1300 | 8.24E-11 | 2.83E-05 | 0.17714 |
| CYP2A6 | cg05910970 | 0.7188 | 0.5742 | 1.85E-09 | 5.98E-04 | 0.14460 |
| CYP2A6 | cg13537471 | 0.7354 | 0.7809 | 5.17E-04 | 1.00E+00 | -0.04554 |
| CYP2A6 | cg19645066 | 0.4930 | 0.5039 | 4.87E-01 | 1.00E+00 | -0.01084 |
| CYP2C19 | cg00051662 | 0.8519 | 0.7391 | 7.65E-12 | 2.72E-06 | 0.11280 |
| CYP2C19 | cg04189838 | 0.9055 | 0.8117 | 1.30E-09 | 4.26E-04 | 0.09384 |
| CYP2C19 | cg20031717 | 0.8001 | 0.7118 | 3.27E-08 | 9.92E-03 | 0.08832 |
| CYP2C19 | cg18564458 | 0.5056 | 0.4586 | 7.29E-08 | 2.17E-02 | 0.04693 |
| CYP2C19 | cg24857560 | 0.8145 | 0.8196 | 2.83E-01 | 1.00E+00 | -0.00513 |
| CYP2C8 | cg12759420 | 0.9117 | 0.9069 | 4.68E-01 | 1.00E+00 | 0.00479 |
